# Antileishmanial Activities of Methanol Extract and Solvent Fraction of *Clematis simensis* Fresen Roots

**DOI:** 10.1155/adpp/9671079

**Published:** 2025-11-26

**Authors:** Amare Megersa, Aschalew Nardos, Tasisa Ketema, Serawit Deyno

**Affiliations:** ^1^ Department of Pharmacy, Dilla University, Dilla, Ethiopia, du.edu.et; ^2^ Department of Pharmacology, Hawassa University, Hawassa, Ethiopia, hu.edu.et; ^3^ Department of Pharmacy, Ambo University, Ambo, Ethiopia, ambou.edu.et; ^4^ Center for Innovative Drug Development and Therapeutic Trials for Africa, Addis Ababa University, Addis Ababa, Ethiopia, aau.edu.et

**Keywords:** antiamastigote, antileishmanial activity, antipromastigote, *Clematis simensis* Fresen roots, *L. aethiopica*, *L. donovani*

## Abstract

**Background:**

Leishmaniasis is a neglected tropical disease affecting 12 million people and risks the lives of 350 million people globally. However, it has limited therapeutic options. *Clematis simensis* Fresen roots are used for the treatment of leishmaniasis in Ethiopian traditional medicine.

**Objective:**

The aim of this study was to evaluate *C. simensis* Fresen roots’ antileishmanial activities and cytotoxic effects.

**Methods:**

In vitro antileishmanial activity of crude extract of *C. simensis* root and its solvent fractions was evaluated against axenic amastigotes and promastigotes of *Leishmania aethiopica* and *Leishmania donovani*. Their cytotoxicity against macrophage cells and red blood cells (RBCs) was evaluated. Median cytotoxic concentration (CC_50_) and median inhibitory concentration (IC_50_) were calculated using computer software GraphPad Prism 9.5.0. Data were expressed as mean ± standard error of the mean.

**Result:**

The crude extract and its hexane, chloroform, and aqueous fractions exhibited antileishmanial activities, with IC_50_ values ranging from 4.43 ± 0.69 to 24.94 ± 4.12 μg/mL against promastigotes and 4.52 ± 1.25 to 34.97 ± 1.77 μg/mL against axenic amastigotes of *Leishmania* parasites. The cytotoxic effects (CC_50_) of the extracts were 63.62 ± 4.06 ≤ CC50 ≤ 246.05 ± 32.84 μg/mL against macrophages, but > 1200 μg/mL against RBC. A selectivity index value greater than 1 indicates selective toxicity against *Leishmania* parasites, whereas a value less than 1 suggests selective toxicity against mammalian cells. In this study, the selectivity indices for the plant extracts ranged from 3.26 to 54.44.

**Conclusion:**

The roots of the plant showed a promising antileishmanial activity that may provide a scientific justification for its use in traditional medicine. Further isolation, identification of active compounds, and establishment of the mechanism of action are warranted.

## 1. Background

Leishmaniasis is a neglected tropical disease caused by *Leishmania* parasites [1]. It affects 12 million people over the world, and 350 million people are at risk of infection [2, 3]. Each year in the United States, a high number of cutaneous and 3500 cases of visceral leishmaniasis (VL) were reported [4]. VL is a deadly disease strongly associated with poverty and poor health care [5]. The Global Burden of Disease Study indicates that neglected tropical diseases (NTDs) were responsible for 62 million disability‐adjusted life years (DALYs), where leishmaniasis was responsible for 774,000 DALYs among NTDs [3]. VL ranked first in terms of disease burden [6]. High global mortality, economic, social, and psychological impact of the diseases is significant, particularly in developing countries [5].

Despite efforts to develop a vaccine for humans, chemotherapy is the main treatment for leishmaniasis [7]. However, few drugs with many drawbacks are available for the treatment of the disease, such as excessively long treatment durations, severe side effects, exorbitant costs, or invasive administration methods requiring prolonged hospitalization, which limit their use in disease‐endemic areas. In addition, their effectiveness is called into question by the increasing number of treatment failures and drug resistance [8]. Therefore, searching for effective, less toxic, and convenient antileishmanial drugs is required.

*Clematis simensis* Fresen (genus: *Clematis*; family: Ranunculaceae) is a flowering plant that has been traditionally used in folk medicine for the treatment of various illnesses due to its medicinal properties. For instance, preparations made from the leaves [9] and a concoction of powdered roots mixed with honey [10] have been applied topically to treat cutaneous leishmaniasis. The aim of the current study was to evaluate the antileishmanial activity of methanol extracts of *C. simensis* Fresen roots and its solvent fractions against *Leishmania donovani* and *Leishmania aethiopica* in vitro.

## 2. Materials and Methods

### 2.1. Chemicals and Reagents

Chemicals and reagents used in the experiment include hexane, methanol (Sisco Research Laboratories, India), chloroform (Carlo Erba, France), distilled water (Leishmania Research and Diagnostic Laboratory, Addis Ababa University), phosphate buffer saline, potato starch, resazurin sodium salt, dimethyl sulfoxide (DMSO) (laborchemikalien GmbH, Germany), Triton X‐114, HCl (Loba Chemie, India), amphotericin B (all from Sigma Aldrich, USA/Germany), tryptophan blue stain (Grand Island, USA), and hydrochloric acid (Loba Chemie, India).

### 2.2. Culture Medium

The culture medium and supplements include minimum essential medium (MEM), heat‐inactivated newborn calf serum, penicillin streptomycin solution, (4‐(2‐hydroxyethyl)‐1piperazine) ethane sulfonic acid), medium‐199 with Earle’s salts, L‐glutamine, penicillin–streptomycin/mL, Roswell Park Memorial Institute, D (+)‐glucose, nutrient agar, calcium chloride, potassium chloride, sodium bicarbonate, sodium chloride (all from Sigma‐Aldrich USA), and defibrillated and heat‐inactivated sheep blood.

### 2.3. Equipment and Supplies

Eppendorf tubes (1.5, 15, and 50 mL), polystyrene sterile tissue culture flasks (25, 50, and 75 mL) (Corning Incorporated, USA), 96‐well flat‐bottom tissue culture plates (Nunc International, USA), analytical balance (Mettler Toledo, Switzerland), autoclave machine (Timo, Italy), biosafety cabinet with UV (Laboculture ESCO class IIA), centrifuges, carbon dioxide incubator (Thermo Electron), digital water bath (Julabo TW20, Thermo Electron), hemocytometer compound microscope and inverted microscope (Olympus and Labomed), lyophilizer (Operon, Korea), table‐top vacuum filter (Micropore, Brazil), micropipettes, multichannel pipettes (Hamilton), microplate reader (Victor3 Perkin Elmer), pipette tips and their rack (Eppendorf), BÜCHI Rota vapor R‐200, heating bath (BÜCHI Heating Bath B‐490), deep freezer, filter paper (Whatman number one), gauze (nylon clothes), mortar and pestle, magnetic stirrer, vortex, separatory funnels, serological pipettes, pH meter, aluminum foil, oven dryer, syringe, and gloves.

### 2.4. Parasite Test Strains, Animal Cells, and Animals

The clinical isolates of *L. aethiopica* (CL‐854/17) and *L. donovani* (VL‐Gr‐1140/23) parasite strains were acquired from the Leishmaniasis Research and Diagnostic Laboratory, Microbiology, Immunology, and Parasitology Department, Addis Ababa University, Ethiopia. Red blood cells (RBCs) and peritoneal macrophages were collected from healthy 7–8‐week‐old Swiss albino mice, while the animals were obtained from the Animal Laboratory, Pharmacology Department, Addis Ababa University, Ethiopia.

## 3. Methods

### 3.1. Plant Material Collection

The roots of *C. simensis* Fresen were collected from Gulele Botanical Garden, located in Gulele sub‐city, Addis Ababa, in the month of December 2022. The plant was authenticated by Mr. Melaku Wondafrash, a senior staff member and botanist at the College of Natural and Computational Sciences at Addis Ababa University. The voucher number AM‐001 was given to the plant specimen and kept in the National Herbarium of Addis Ababa University for future reference.

### 3.2. Preparation of Crude Extracts

The collected roots of *C. simensis* Fresen were dried under shade within the first 3 weeks and ground to a coarse powder with the aid of a mortar and pestle. Then, 1350 g of the powder was macerated with methanol (1:5) in volumetric flasks for 3 days. To maximize the solvent penetration and solubility of the active ingredients, the macerate was agitated occasionally. After 3 days of maceration, the extracts were filtered with muslin gauze several times and then with Whatman NO‐1 filter paper. By adding a fresh solvent to the mark, the maceration and filtration were repeated twice in order to maximize the yield. The combined filtrates were concentrated using a rotary evaporator at 40°C and dried in an oven at temperatures of 30°C. Finally, the total yield of the dry extract was determined, and the percent yields were calculated using the following formula:

(1) yield %=weight of extract obtained weight of extracted plant sample×100.

### 3.3. Preparation of Solvent Fractions

The dried methanol extract of the plant was fractionated into hexane, chloroform, and aqueous fractions, respectively [11]. methanol extract (21.25 g) was transferred to a separatory funnel, reconstituted in 250 mL of water, and vigorously washed with hexane (250 mL) in a 1000 mL separating funnel (3 times), followed by equal volumes of chloroform (3 times) until the extracting solvent became colorless. The remaining portion after successive fractionation was an aqueous fraction. Additional methanol extract (21.25 g) of the plant was fractionated in a similar manner. The hexane and chloroform fractions were concentrated using a rotary evaporator at a temperature of 40°C and dried in an oven at 30°C, whereas the aqueous fraction was dried using a lyophilizer at reduced pressure and temperature. The dried fractions were weighed, packed in a separate vial, and stored in a deep freezer at −20°C until used for the procedure.

Finally, 84.98 g (6.29%) of the crude methanol extract of the *C. simensis* Fresen, having a deep yellow, semisolid mass nature, was obtained. The yields of n‐hexane, chloroform, and aqueous fractions of 42.49 g of methanol extract were 36 g (84.72%), 4.69 g (11.03%), and 1.89 g (4.45%), respectively.

### 3.4. Mice Peritoneal Macrophage Collection

The macrophage cells were collected from 6‐ to 8‐week‐old Swiss albino mice of either sex according to a previously described protocol [12], with minor modification. Two milliliters of 2% potato starch solution were administered to each mouse, intraperitoneally, using an insulin syringe. Post 48 h of administration, each mouse was sacrificed with chloroform; the skin under the abdominal cavity was swabbed with 70% ethanol and opened with sterile surgical scissors. The sterile solution of ice‐cold phosphate‐buffered saline (PBS) supplemented with 3% HINBCS (10 mL) was injected into the abdominal cavity of each animal. Then, the abdomen was massaged for approximately 2 minutes, and macrophage cells were collected by withdrawing 6–8 mL of peritoneal exudate from each mouse using a 10 mL syringe. The obtained exudates were centrifuged at 450 g (1500 rpm) for 10 min, where the supernatant was discarded while pellets were resuspended in cold MEM supplemented with 10% HINBCS, 25 mM HEPES, 2 mM L‐glutamine, 100 IU penicillin, and 100 μg streptomycin/mL. The cells were counted using a hemocytometer and adjusted to 3 × 10^6^ cells/mL.

### 3.5. Culture Conditions

#### 3.5.1. Promastigote Culture

The clinical isolates of *L. donovani* and *L. aethiopica* were grown in Novy–MacNeal Nicolle (NNN) media supplemented with Locke’s solution in 10 mL vials. Then, the logarithmic growth phases of each parasite were subcultured and grown in a separate 25 mL tissue culture flask containing RPMI‐1640 medium supplemented with 10% heat‐inactivated fetal calf serum (HIFCS) and 100 IU/mL penicillin/100 μg/mL streptomycin solution. The cultures were maintained at 22°C for *L. aethiopica* and 26°C for *L. donovani* following previously described methods [12–14].

#### 3.5.2. Amastigote Culture

The axenic cultures were laid out according to the methods described for *L. aethiopica* [15] and *L. donovani* [16], with minor modifications. Promastigotes in the late stationary phase were centrifuged and resuspended in medium 199 containing Hank’s balanced salts supplemented with 20% HIFCS, 2 mM L‐glutamine, and penicillin–streptomycin solution (50 IU/mL/50 μg/mL), where the pH was adjusted to 5.5 (for both strains) with 0.10 N HCl. Then, the cells were incubated in a humidified 95% CO_2_ incubator for 7 days at 31°C (for *L. aethiopica*) or 37°C (for *L. donovani*).

### 3.6. Antileishmanial Activity Assay

#### 3.6.1. Antipromastigote Assay

The antipromastigote activities of the methanol extract of *C. simensis* Fresen and its fractions were conducted according to a method described previously by [17, 18]. A serially diluted concentration of each extract (500–3.90625 μg/mL) was prepared in complete RPMI‐1640 medium. One hundred microliters of each serial dilution were added to separate wells of a 96‐well microtiter plate, followed by 100 μL of the promastigote parasite suspensions in logarithmic growth stages (3.00 × 10^6^ cells/mL of *L. aethiopica* or *L. donovani*). Then, the contents in the microtiter plates were incubated for 3 days at 22°C (for *L. aethiopica*) and 26°C (for *L. donovani*). Post 68 h of incubation, 20 μL of 10% resazurin sodium salt (0.125 mg/mL, pH = 7.2) was added to each well and permitted to rest for 4 h. Then, fluorescence intensity was measured using a multilabel reader at an excitation wavelength of 544 nm and an emission wavelength of 590 nm. Serially diluted concentrations of AMB (7.5–0.05859 μg/mL) and media plus 1% DMSO were used as positive and negative controls, respectively. Serial concentrations of all test substances and controls were prepared in twofold dilutions with each test concentration in triplicate. The antipromastigote activities of each test substance were expressed as a percentage of parasite inhibition and determined according to the following formula:

(2) inhibition %=100100−absorbance in duplicate drug wells−average blank wellsaverage absorbance in control wellsNC−average blank wells×.

NC = average absorbance of negative control, blank wells = media only, absorbance in duplicate drug wells = absorbance of sample.

#### 3.6.2. Antiamastigote Test.

The antiamastigote activities of the extract and fractions were determined as a previously established method [21]. In separate wells of 96‐well microtiter plates, 100 μL of serially diluted concentrations of extracts (500−3.90625 μg/mL), AMB (7.5−0.05859 μg/mL), and negative control (1% DMSO+ medium) and then 100 μL of parasite suspension (3.00 ×106 cells/mL of axenic amastigotes) were added. Then, the plates were incubated in a humidified 95% CO2 incubator for 72 h at 31°C (for *L. aethiopica*) or 37°C (for *L. donovani*). After 68 h of incubation, 20 μL of 10% resazurin solution was added to each well’s contents, and fluorescence intensity was measured after a total incubation time of 72 h using a multilabel reader at emission and excitation wavelengths of 590 and 544 nm, respectively. Serial concentrations of extracts and controls were prepared in twofold dilutions with each test concentration in triplicate.

### 3.7. Cytotoxicity Assay

#### 3.7.1. Hemolysis Test

Tests for hemolytic effects of the methanol extract of *C. simensis* Fresen and its fraction were done as previously described [19]. Four milliliters of the whole blood obtained by cardiac puncture of mice with a sterile syringe was transferred to a heparinized tube. Two milliliters of blood and 8 mL of PBS solution (pH 7.2) were poured into a 15 mL Eppendorf tube. The mixture was mixed well and then centrifuged for 10 min at 1000 × g. The supernatant was pipetted off with serological pipettes, while the resulting pellet (1 mL) was mixed with a sufficient quantity of PBS solution (pH 7.2) to obtain 50 mL of a 2% red blood cell suspension required for the procedure. Two hundred microliters of the suspension were poured into 1.5 mL Eppendorf tubes prefilled with 200 μL serially diluted concentrations of extracts (1200–9.375 μg/mL) and standard drugs; AMB (7.5–0.05859 μg/mL). PBS solution was used as a dilution medium. Triton X‐114 (5 μL/mL) in PBS solution and PBS solution plus 1% DMSO were used as positive and negative controls, respectively. The Eppendorf tube contents were mixed well and incubated for 2 h except for the Triton X‐114‐containing tube, which was incubated for 30 min at 37°C in the incubator. After the incubation period had elapsed, the Eppendorf tubes were centrifuged at 1000 × g for 10 min. Then, 100 μL of the supernatant was transferred from each tube into a separate well of microtiter plates (96‐well) carefully without affecting the sediment. Finally, the absorbance of the supernatants was measured at 540 nm using the Victor3 Multilabel Reader.

The hemolytic effects of each test substance were expressed as a percentage using the following formula:

(3) hemolysis %=absorbance of samples in  well−average blank wellsTritonX−114average absorbance in a well−average blank wells×100.

#### 3.7.2. Mouse Macrophage Viability Test

The cytotoxic effects of the methanol extract and fraction of *C. simensis* Fresen against the isolates of mouse peritoneal macrophages were determined using a previously described method [20] with minor modifications. One hundred microliters of macrophage cell suspension (3.00 × 10^6^ cells/mL) in complete MEM medium were seeded in each well of 96‐well microtiter plates. Serially diluted concentrations of extracts (1200–9.375 μg/mL) and positive controls; AMB (7.5–0.05859 μg/mL) and negative controls (1% DMSO + medium), each in complete MEM, were added into separate wells of the plates. The plates were incubated for 3 days at 37°C, 5% CO_2_ and 95% humidity in a CO_2_ incubator. After 68 h of incubation, 20 μL of a 10% resazurin solution was added to each well and allowed to rest for 4 h. Then, the fluorescence intensity of each well was measured using a multilabel reader at emission and excitation wavelengths of 590 and 544 nm, respectively. Serial concentrations of test groups were prepared in twofold dilutions, with each test concentration in triplicate.

Cytotoxic effects of each test groups against mouse macrophage cells were expressed as a percentage of macrophage viability calculated using the following formula:

(4) cell viability %=absorbance in duplicate drug wells−average blankwells average absorbance control wells−average blankwells×100.

The antiamastigote activities were expressed as a percentage of parasite inhibition and determined according to the following formula:

(5) inhibition %=100100−absorbance in duplicate drug wells−average blank wellsaverage absorbance control wellsNC−average blank wells×.

### 3.8. Selectivity Index (SI)

The SI of the controls, extract, and its solvent fraction were determined from their CC_50_ value against mammalian cells (mice macrophages and RBCs) and their corresponding IC_50_ value against axenic amastigotes as previously described [22]. The selectivity of the extract and standards in killing parasites as opposed to mammalian cells was determined by using the following formula:

(6) selective index SI=CC50 of a macrophage or red blood cellsIC50 of parasite.

### 3.9. Statistical Analysis

The IC_50_ of antipromastigote and anti‐amastigote activities were calculated from sigmoidal dose–response curves (concentration vs. percentage inhibition curve). Hemolytic activity against RBCs and cytotoxicity of the extract against mouse macrophages were calculated from sigmoidal dose–response curves of percent cell viability. Data were expressed as mean ± standard error of the mean (SEM). GraphPad Prism 9.5.0 computer software (GraphPad Software, Inc., CA, USA) was used for all data analyses. In addition, the SI was determined from the ratio between CC_50_ and IC_50_. All analyses were carried out with a significance level of 5%.

## 4. Results

### 4.1. Antileishmanial Activity

The crude methanol extract of *C. simensis* Fresen and its hexane, chloroform, and aqueous fractions showed antileishmanial activity against clinical isolates of promastigotes and axenic amastigotes of *L. donovani* and *L. aethiopica* with varying IC_50_ values. The hexane fraction showed the strongest antileishmanial activity, with 4.43 ± 0.69 ≤ IC_50_ ≤ 7.16 ± 0.62 μg/mL against promastigotes and axenic amastigotes of *L. aethiopica*. On the other hand, the aqueous fraction (22.63 ± 2.88 ≤ IC_50_ ≤ 24.94 ± 4.12 μg/mL) exhibited the lowest activities against the promastigotes and amastigotes of each *Leishmania* species (Tables 1 and 2).

**Table 1:** The IC_50_ of test substances against promastigotes of *L. donovani* and *L. aethiopica*

Test substance	*L. donovani*		*L. aethiopica*	
	IC_50_ (μg/mL)^a^	*R* ^2^	IC_50_ (μg/mL)^a^	*R* ^2^
MET–CS	6.35 ± 0.68	0.987	5.55 ± 0.41	0.996
HEX–CS	4.43 ± 0.69	0.979	7.16 ± 0.62	0.991
CHL–CS	5.33 ± 0.32	0.997	15.14 ± 0.50	0.998
AQU–CS	24.94 ± 4.12	0.967	22.63 ± 2.88	0.977
AMB (standard drug)	0.05 ± 0.0.015	0.9423	0.0452 ± 0.011485	0.8924
Medium alone (blank)	0.00^d^		0.00^d^	
1% DMSO + medium (NC)	0.00^d^		0.00^d^	

*Note:* The values are expressed as mean ± SEM; *n* = 3; MET–CS: crude methanol extract of roots of *Clematis simensis* Fresen; HEX–CS, CHL–CS, AQU–CS: are hexane, chloroform, and aqueous fractions, respectively; DMSO: dimethyl sulfoxide. *R* ^2^: regression coefficient; AMB: amphotericin B.

Abbreviation: NC, negative control.

^a^Effective concentration required to achieve 50% growth inhibition in μg/mL.

^d^No effect.

**Table 2:** The IC_50_ of test substances against axenic amastigotes of *L*. *donovani* and *L. aethiopica*.

Test substance	*L. donovani*		*L. aethiopica*	
	IC_50_ (μg/mL)^a^	*R* ^2^	IC_50_ (μg/mL)^a^	*R* ^2^
MET–CS	7.94 ± 1.15	0.976	17.42 ± 1.15	0.989
HEX–CS	4.52 ± 1.25	0.995	11.36 ± 1.38	0.979
CHL–CS	8.77 ± 1.64	0.973	19.37 ± 1.18	0.992
AQU–CS	34.97 ± 1.77	0.996	29.59 ± 1.88	0.993
AMB (standard drug)	0.049 ± 0.015	0.9403	0.038 ± 0.009	0.9627
Medium alone (blank)	0.00^d^		0.00^d^	
1% DMSO + medium (NC)	0.00^d^		0.00^d^	

*Note:* The values are expressed as mean ± SEM; *n* = 3; MET–CS: crude methanol extract of roots of *Clematis simensis* Fresen; HEX–CS, CHL–CS, AQU–CS: are hexane, chloroform, and aqueous fractions, respectively; DMSO: dimethyl sulfoxide. *R* ^2^: regression coefficient; AMB: amphotericin B.

Abbreviation: NC, negative control.

^a^Effective concentration required to achieve 50% growth inhibition in μg/mL.

^d^No effect.

### 4.2. Cytotoxic Effects

The cytotoxic effects (CC50) of methanol extract of *C. simensis* Fresen and its solvent fractions against mouse RBCs and peritoneal mouse macrophages were presented. All solvent fractions and crude extract showed CC50 > 1200 μg/mL against mouse RBCs. The crude methanol extract and its hexane, chloroform, and aqueous fractions revealed 8.89 ± 0.60%, 5.48 ± 1.52%, 5.97 ± 0.79%, and 5.76 ± 1.14% of hemolysis at maximum test concentration (1200 μg/mL), respectively. On the other hand, the chloroform fraction revealed the highest cytotoxic effect against macrophage cells (Table 3). The crude methanol extract and its solvent fractions showed selective toxicity toward *Leishmania* parasites with 3.26 ≤ SI ≤ 54.44 and SI ≥ 34.32 against macrophage cells and RBCs, respectively (Tables 4 and 5).

**Table 3:** The CC_50_ of test substances against mouse macrophages and red blood cells.

Test substance	Against macrophage cells		Against red blood cells	
	CC_50_ (μg/mL)^d^	*R* ^2^	CC_50_ (μg/mL)^a^	*R* ^2^
MET–CS	65.62 ± 4.06	0.994	> 1200	—
HEX–CS	246.05 ± 32.85	0.962	> 1200	—
CHL–CS	63.07 ± 7.69	0.976	> 1200	—
AQU–CS	159.55 ± 28.15	0.952	> 1200	—
AMB (standard drug)	8.82 ± 6.9	0.8440		
Medium alone (blank)	0.00^d^		0.00^d^	
1% DMSO + medium (NC)	0.00^d^		0.00^d^	

*Note:* The values are expressed as mean ± SEM; *n* = 3; MET–CS: crude methanol extract of roots of *Clematis simensis* Fresen; HEX–CS, CHL–CS, AQU–CS: are hexane, chloroform, and aqueous fractions; respectively; DMSO: dimethyl sulfoxide. *R* ^2^: regression coefficient; AMB: amphotericin B.

Abbreviation: NC, negative control.

^a^Effective concentration required to kill 50% of animal cell lines in μg/mL.

^d^No effect.

**Table 4:** Selectivity index of methanol extract of *Clematis simensis* and its fraction against macrophage cells.

Test substance	Selectivity index (SI), CC_50_/IC_50_ (μg/mL)^a^	
	*L. donovani*	*L. aethiopica*
MET–CS	8.26	3.77
HEX–CS	54.44	21.61
CHLO–CS	7.19	3.26
AQU–CS	4.59	5.43
AMB (standard drug)	220.5	232.11

*Note:* The values are expressed as mean ± SEM; *n* = 3; MET–CS: crude methanol extract of roots of *Clematis simensis* Fresen; HEX–CS, CHL–CS, AQU–CS: are hexane, chloroform, and aqueous fractions, respectively; DMSO: dimethyl sulfoxide. *R* ^2^: regression coefficient; AMB: amphotericin B.

Abbreviation: NC, negative control.

^a^The value selectivity index is calculated as the ratio of the CC_50_ of a mammalian cell to its respective IC_50_ against a parasite.

**Table 5:** Selectivity index of methanol extract of *Clematis simensis* and its fraction against red blood cells.

Test substance	Selectivity index (SI), CC_50_/IC_50_ (μg/mL)^a^	
	*L. donovani*	*L. aethiopica*
MET–CS	> 151.13	> 68.89
HEX–CS	> 265.49	> 105.63
CHLO–CS	> 136.83	> 61.95
AQU–CS	> 34.32	> 40.55
AMB (standard drug)	180	232.12

*Note:* The values are expressed as mean ± SEM; *n* = 3; MET–CS: Crude methanol extract of roots of *Clematis simensis* Fresen; HEX–CS, CHL–CS, AQU–CS: are hexane, chloroform, and aqueous fractions, respectively; DMSO: dimethyl sulfoxide. *R* ^2^: regression coefficient; AMB: amphotericin B.

Abbreviation: NC, negative control.

^a^The value selectivity index is calculated as the ratio of the CC_50_ of a mammalian cell to its respective IC_50_ against a parasite.

## 5. Discussion

In this study, the in vitro antileishmanial activity of crude (methanol) extracts of *C. simensis* Fresen roots and its solvent fractions against the promastigotes and axenic amastigotes of *L. donovani* and *L. aethiopica* was investigated.

The organic solvent fractions showed more antileishmanial activities than the aqueous fraction. This may be due to the preferential solubility of most secondary metabolites in organic solvents rather than in water. The crude extracts showed the highest antipromastigote activity against *L. aethiopica* as compared to solvent fractions. This may be due to the synergistic effects of various active compounds that are present in the crude extracts. On the other hand, the hexane fractions showed the highest antileishmanial activities against promastigotes of *L. donovani* and axenic amastigotes of *Leishmania* species. The antileishmanial activities of the crude extracts and hexane and chloroform fractions were comparable against promastigotes and axenic amastigotes of *L. donovani*, but the crude extracts and hexane fractions showed more activity against promastigotes of *L. aethiopica* than their axenic counterparts. This may be due to the variability of *Leishmania* species.

The IC_50_ of the plant extracts ranged from 89 to 501 and 91 to 779 times that of amphotericin B against the promastigotes and axenic amastigotes, respectively, suggesting that the antileishmanial activities of the root extracts of the plant were much lower than activities shown by the standard drug.

The in vitro antileishmanial activities of the plant extracts and standards were interpreted based on the following criteria: IC_50_ ≤ 5 μg/mL: pronounced activity; 5 < IC_50_ ≤ 20 μg/mL: good activity; 20 < IC_50_ ≤ 30 μg/mL: moderate activity; 30 < IC_50_ ≤ 64 μg/mL: low activity; IC_50_ > 64 μg/mL: inactive [23]. Accordingly, the hexane fractions showed pronounced activities against *L. donovani* and good activities against *L. aethopica* irrespective of their developmental stages. The crude extracts and chloroform fractions demonstrated good activity, while the aqueous fractions showed low to moderate antileishmanial activities (Tables 1 and 2). On the other hand, the susceptibility of *Leishmania* parasites toward amphotericin B was almost equal regardless of the developmental stages of the parasites.

*Leishmania* species have dimorphic forms of life cycle: an extracellular promastigote and an infective intracellular amastigote, which reside in sand fly and mammalian tissues, respectively [24]. Therefore, antileishmanial activities of chemotherapeutic agents need to be conducted against amastigote stages as confirmatory to antipromastigote activity. As amastigotes obtained from infected mammals are often present in insufficient quality and quantity, two alternative methods are employed to transform promastigotes to amastigotes, including axenic amastigotes and macrophage‐derived or intracellular amastigotes for in vitro studies [25]. Axenic amastigotes were employed in our current antileishmanial study of *Clematis simensis*.

Using axenic amastigotes in evaluating the antileishmanial activity of a natural compound has several merits and demerits. As compared to intracellular amastigotes, it is easily manipulated, quantification of the bioactive agent’s effect is rapid and cheap, and the model does not require laboratory animals [26]. However, for compounds that interfere with the parasite–host interaction [27] or exhibit antileishmanial activities through the stimulation of the immune system, intracellular‐based assays are a good indicator of drug effect [28]. Therefore, future studies are warranted to explore the effects of plant extracts on intracellular amastigotes in order to gain a deeper understanding of their biological activity and therapeutic potential.

The in vitro toxicity level of active compounds was appreciated based on the following criteria: < 10 μg/mL: very strong cytotoxicity, 10–100 μg/mL strong cytotoxicity, and 100–500 μg/mL moderate cytotoxicity [29]. In addition, a SI value > 1 is considered to be selective against the *Leishmania* parasites, and < 1 is considered as selective against mammalian cells [29]. The crude extracts and all fractions exhibited moderate cytotoxic effects (63.07 ± 7.69 ≤ CC_50_ < 246.05 ± 32.85 μg/mL) against peritoneal mouse macrophages and no cytotoxic effects against RBCs (CC_50_ > 1200 μg/mL). Similarly, the crude extract of *C. simensis* and all fractions were selective against both strains of *Leishmania.* The hexane fractions exhibited the highest selectivity indices compared to the other extracts (Tables 4 and 5). The combination of its superior activity and elevated selectivity indices indicates that further bioactivity‐guided fractionation and compound isolation from this fraction are warranted.

The *Clematis* genus has a wide range of chemical compounds, potentially offering various therapeutic benefits. It contains a numerous mix of ingredients, including triterpenes, flavonoids, lignans, coumarins, and more. However, triterpenoid saponins, flavonoids, and lignans constitute major classes of chemical constituents [30]. Among the triterpenoid saponins, oleanolic acid has been isolated from species like *Clematis ganpiniana* and *Clematis apiifolia*, while β‐hederin and α‐hederin have been isolated from *Clematis tangutica, Clematis ganpiniana, Clematis parviloba, and Clematis tibetana* [30] in the previous studies. The genus is also rich in flavonoids; apigenin can be found in *Clematis viornae* L., *Clematis trichotoma* Nakai, and *Clematis terniflora*, while quercetin is present in *Clematis stans* and *Clematis rehderiana* [12]. Many other *Clematis* species contain additional flavonoids like luteolin and rutin [30, 31]. Beyond this chemical diversity, anemonin, a phenolic compound, has been found in *Clematis angustifolia, Clematis apiifolia*, *Clematis crassifolia, Clematis chinensis, Clematis erecta*, *Clematis flammula*, and *Clematis hirsuta* [30–32].

Anemonin, a natural product isolated from the fresh roots of *Ranunculus multifidus* Forsk [33], demonstrated antileishmanial activities against *L. donovania* and *L. aethiopica*. Oleanolic acid, a triterpenoid isolated from olive leaf extract, exhibited antileishmanial activity against amastigotes of *Leishmania infantum* (IC_50_ = 0.999 ± 0.089 μg/mL) and *Leishmania amazonensis* (IC_50_ = 3.040 ± 0.505 μg/mL) [22]. Other triterpenoid saponins, such as β‐hederin and α‐hederin isolated from the roots of *Hedera helix*, showed antileishmanial activities against promastigotes (IC_50_ = 12.0 ± 0.05, 13.6 ± 0.08 μM) and amastigotes (IC_50_ = 0.25 ± 0.05, 0.35 ± 0.3 μM) of *L. infantum*, respectively [34]. Apigenin, a naturally occurring plant flavone present in common fruits and vegetables, has revealed in vitro and in vivo antileishmanial activity against *L. infantum* and *L. amazonensis* [21]. A flavonoid compound known as quercetin also showed antileishmanial activity against amastigotes (IC_50_ = 21 ± 2.5 μM) and promastigotes (IC_50_ = 25 ± 0.7 μM) of *Leishmania Viannia braziliensis.* Other flavonoid compounds, luteolin, isolated from the leaves of *Vitex negundo* [35], and rutin, isolated from natural products [36], exhibited antileishmanial activities. The leaves of extracts of *C. simensis* also demonstrated antileishmanial activities in the previous study [37].

This suggested that the antileishmanial activities of the root extracts of *C. simensis* Fresen were attributed to the individual or combined effects of flavonoids, phenolic compounds, triterpenoid saponins, and other chemical constituents present in the plant.

## 6. Conclusion

The result of the present study demonstrated the in vitro antileishmanial activity of the crude (methanol) extract of *C. simensis* Fresen and its solvent fraction against *L. donovani* and *L. aethiopica*. The finding might provide a scientific justification for the use of *C. simensis* Fresen root extracts for the treatment of leishmaniasis in the traditional medicine of Ethiopia. Isolation and identification of active ingredients responsible for antileishmanial activity are required. Further in vivo study and the mechanism of antileishmanial activity of the plant are also required.

**Nomenclature**
AMB: Amphotericin B
CC5_0_
: 50% cytotoxic concentration
CL: Cutaneous *Leishmania*
CR1: Complement Receptor1
CR3: Complement Receptor3
DALYS: Disability‐adjusted life years
DCL: Disseminated cutaneous *Leishmania*
DMSO: Dimethyl sulfoxide
HEPES: (4‐(hydroxyethyl)1‐piperazine ethane sulfonic acid)
HINBCS: Heat‐inactivated newborn calf serum
IC_50_
: Effective concentration that which inhibits 50% of the cells
M‐199: Medium‐199 with Earle’s salts
MEM: Minimum essential medium
MF: Miltefosine
PBS: Phosphate‐buffered saline
PK/PD: Pharmacokinetics and pharmacodynamics
PKDL: Post‐Kala‐Azar dermal leishmaniasis
RPMI1640: Roswell Park Memorial Institute‐1640 Media
VL: Visceral *Leishmania*.

## Ethics Statement

Ethical clearance for this study was obtained from the College of Medicine and Health Sciences at Hawassa University (Reference Number: ERB/158/14/2023, March 2023). Informed consent was secured from all patients for parasite isolates. All procedures complied with the WMA Declaration of Helsinki and local regulations for human research. The Ethics Review Board ensured compliance with regulations for experimental animals, confirming that all animal handling procedures complied with European Directive 2010/63/EU.

## Disclosure

The funder has no involvement in the study design or submission of the study for publication.

## Conflicts of Interest

The authors declare no conflicts of interest.

## Author Contributions

A.M. and T.K. conceptualized the work, wrote the original draft, investigated the experiment, and collected data. A.N. supervised, revised, and edited this work. S.D. revised and finally edited it.

## Funding

Hawassa University through its thematic research grant supported this research.

## Data Availability

Data supporting the results of this study will be available upon request.
